# On Vaccine Inoculation

**Published:** 1802-03

**Authors:** 


### *On VACCINE INOCULATION.*

### By Dr. COGAN.

THE following extracts from a letter on Vaccine Inoculation, communicated by ſeveral medical gentlemen in Rotterdam to the Agent of the National Inſtitution, appear to me not not unworthy the attention of the Engliſh reader, as their experiments were made in a manner peculiarly ſatisfactory, and as ſome parts of the information are both novel and intereſting.

In the month of October, 1799, Mr. Davids, a practitioner in Rotterdam, in conjunction with M. Duvigneau, made their firſt experiment with matter received from Dr. Walker, which ſucceeded to the utmoſt extent of their wiſhes, and encouraged theſe gentlemen and ſeveral others, animated by their ſucceſs, to inveſtigate the ſubject more minutely. They made ſeveral attempts to procure matter of the Cow-pox both from France and England; but theſe proving abortive, from a concurrence of circumſtances, Mr. Davids undertook a journey to Paris, not only to procure matter, but to obtain information concerning whatever related to the practice. Upon his return he encouraged our deſigns, by aſſurances that the experience of the practitioners at Paris perfectly coincided with the reports received out of England. But neither the Vaccine matter which he brought with him, nor that ſent to us from England, proved ſucceſsful. At length we received four lancets, charged with the matter from the Inoculating Hoſpital in London, and, at the ſame time, an infected thread from Dr. Nowel, at Bologne. With theſe, ſeveral children were inoculated, and we had the ſatisfaction to ſee that it ſucceeded with three in the moſt perfect manner; which cheriſhed the joyful proſpect, that the *Vaccina*, having once taken root among us, would in proceſs of time ſpread itſelf over the country, and annihilate one of the moſt dreadful calamities to which humanity is ſubject.

To detail all the circumſtances attending their various experiments, would be an unneceſſary repetition of what has been ſo repeatedly communicated to the public, I ſhall therefore confine myſelf to a few of the moſt intereſting.

"We found (ſays the writer) that the matter for inoculation muſt be taken as ſoon as it appears in the wound; and that it was not proper to wait longer than three days, generally from the eighth to the tenth; for as ſoon as it became thick, and of the conſiſtency of *pus*, we found either that the inoculation did not take place, or it produced a ſpurious pock, which was no ſecurity againſt the natural infection.

"Though, in general, the progreſs of the wound was perfectly ſimilar to that with Variolous matter, yet we found that it was much ſlower in ſome caſes; ſo that the wound did not exhibit any marks of activity before the ninth, tenth, and even the fourteenth day after the inciſion.

"In order to obſerve the ſimilarities and differences more minutely, we inoculated with the natural Small-pox at the ſame time, time, and under the fame circumftances; and we found that the fwelling, and production of matter in the wounds, were fo fimilar both in appearance and in time, that the moft experienced inoculator could not difcover any difference until the tenth or eleventh day.

"We obferved that the Cow-pox did not require, either before or after inoculation, any change of regimen, which was the cafe with the Small-pox.

"About the eighth day (in fome earlier, in others later) moft children who were able to exprefs their feelings, complained of a degree of pain under the axillæ; and this was followed, on the ninth or tenth, with a great quicknefs of pulfe, and rednefs on the countenance, which, in fome inftances, was attended with a rednefs over the whole body. Thefe flight appearances of fever were accompanied with a lofs of appetite and general indifpofition; but thefe are not to be compared with the effects of Variolous matter at the fame period.

"Of the numbers that were inoculated, fome of the children were weak and fickly; one was fubject to fits from its birth; but in none of thefe were their diforders increafed; and in the child fubject to convulfions, we found that thefe were much lefs during the inflammation of the wound. In fome that had cutaneous difeafes, fuch as the ring-worm, &c. the eruptions either totally difappeared, or were much diminifhed. In thefe fubjects the *cruft* generally fell off the earlieft, and left an obftinate but harmlefs ulcer.

"A perfon who had been inoculated with the Cow-pock four days, indicated fymptoms of the natural infection; and thefe put on the appearance of the *confluent* kind; the fkin was covered with eruptions; but, in proportion as the Vaccina advanced, thafe eruptions became *diftinct*, and diminifhed aftonifhingly in number. The efchar, or cruft, remained a length of time after the difeafe had terminated.

"In twenty cafes Variolous inoculation was fuper-added, without producing the difeafe. In all thefe, the wounds were inflamed on the firft or fecond day; on the third they were more inflamed and fwollen; and, in fome inftances, to fuch a degree that we began to fufpect a general infection would fucceed; but thefe were, on the fourth day, lefs inflamed and fwollen, and they difappeared on the fifth.

"One of our inoculated patients was placed by its father in bed with another child that had the natural Small-pox to an alarming degree, and continued in this fituation for feveral days without experiencing the leaft effect. One of our furgeons, Mr. Philipeau, whofe daughter was *vaccined* under our infpection, tion, permited the child to embrace a patient in the *confluent* difeafe, juft at the period of maturation. The patient died, but the child received no injury.

"We have obferved, in general, that a fingle pock manifefted itfelf upon each wound; but, in fome inftances, we obferved one or two others in the vicinity. In one cafe we counted about feventy fmall feftering pocks furrounding the large one, and fcarcely to be diftinguifhed before they dried up from appearances in the Variolous Inoculation. The fcale of the large pock was of a much darker colour than the others; thefe fell off together, left an inflamed wound which yielded pus, and continued for a long time; but was at length completely cured.

"In one patient we remarked, that after the twelfth day, when the cruft fell off, leaving a large quantity of matter in the wound, feveral pocks appeared upon one cheek. Whether or not thefe were communicated by the mother, who by putting on the cap after dreffing the wound, might have infected that fide of the cheek where fhe faftened the band, we could not afcertain. It is certain, that in another child, where no fuperinfection could take place, Vaccine eruptions, about five or fix, appeared on the extremities; and in a third, a fmall number was fpread over the body. We inoculated with the matter taken from the cheek of the firft, which produced the genuine Vaccine pock. A woman who dwelt in the fame houfe with this child, and who had had the Small-pox in early life, by frequently embracing the child, had Vaccine eruptions in her face, manifeftly of the fpurious fort.

"Seven days after the inoculation, one of the patients caught the meafles, which were at that time prevalent. The pock already formed was checked in its progrefs for the fpace of fourteen days, excepting that it became fomewhat enlarged, until the meafles were terminated, which was on the twenty-firft day after inoculation. The Cow-pox now began to acquire activity, formed the areola, and became dry on the twenty-fourth. The meafles were of a very unfavourable kind; and it appeared to us highly probable that the patient would have fallen a victim to its malignancy without the aid of the Cow-pox, which, though apparently ftationary, acted as a ftimulant to the fyftem. In this it refembled the natural Small-pox, and fuggefted to us an idea of the poffibility that the Small-pox might, in its origin, have been a *Vaccine* malady; which, in confequence of various changes and modifications, degenerated in the human body until it acquired its known malignancy. Farther difcoveries may enable us to confirm or reject this idea.

"We

"We have been indefatigable in our Inquiry, whether the Cow-pox was a malady known among the cattle in the Netherlands; but have not as yet acquired satisfactory information.

"Signed, TH. F. VAN OPDORP.
L. DAVIDS.
F. H. GRAM.
I. F. DUVIGNEAU.
C. VAN HATTEM."

